# The value of autopsy in preterm infants at a Swedish tertiary neonatal intensive care unit 2002–2018

**DOI:** 10.1038/s41598-021-93358-7

**Published:** 2021-07-08

**Authors:** Alice Hoffsten, Laszlo Markasz, Katharina Ericson, Leif D. Nelin, Richard Sindelar

**Affiliations:** 1 Department of Women’s and Children’s Health, Uppsala University, Uppsala, Sweden; 2 Neonatal Intensive Care Unit, Uppsala University Children’s Hospital, Uppsala, Sweden; 3 Department of Pathology, Uppsala University Hospital, Uppsala, Sweden; 4 Nationwide Children’s Hospital, Ohio State University College of Medicine, Columbus, OH USA

**Keywords:** Health care, Medical research, Signs and symptoms

## Abstract

Reliable data on causes of death (COD) in preterm infants are needed to assess perinatal care and current clinical guidelines. In this retrospective observational analysis of all deceased preterm infants born < 37 weeks’ gestational age (n = 278) at a Swedish tertiary neonatal intensive care unit, we compared preliminary COD from Medical Death Certificates with autopsy defined COD (2002–2018), and assessed changes in COD between two periods (period 1:2002–2009 vs. period 2:2011–2018; 2010 excluded due to centralized care and seasonal variation in COD). Autopsy was performed in 73% of all cases and was more than twice as high compared to national infant autopsy rates (33%). Autopsy revised or confirmed a suspected preliminary COD in 34.9% of the cases (23.6% and 11.3%, respectively). Necrotizing enterocolitis (NEC) as COD increased between Period 1 and 2 (5% vs. 26%). The autopsy rate did not change between the two study periods (75% vs. 71%). We conclude that autopsy determined the final COD in a third of cases, while the incidence of NEC as COD increased markedly during the study period. Since there is a high risk to determine COD incorrectly based on clinical findings in preterm infants, autopsy remains a valuable method to obtain reliable COD.

## Introduction

The autopsy has been fundamental to understand anatomy and pathophysiology in medicine and neonatology. Despite proactive obstetric and neonatal care, prematurity remains the most common worldwide cause of death (COD) among children under the age of 5 years^1,2^. As autopsy is “the gold standard” for accurately determining COD, autopsy contributes to the continued development of neonatal care and possibly improved survival in preterm infants^3,4^.

When reporting causes of death, it is customary to use the International Classification of Diseases (ICD) by the WHO^5^. To assign prematurity as the COD is not recommended by the WHO, unless no other COD can be confirmed. Despite these recommendations, there has been a continued use of prematurity as COD instead of defining the specific COD^6–8^. In mortality reports, ICD-codes are grouped into categories to provide coherent descriptions, although, classification systems often differ between reports and/or countries. Based on Wigglesworth’s classification of perinatal mortality^9^, the International Collaborative Effort (ICE) offered a classification method for infant mortality in 1989 to enable international comparisons^10^. Still, reports continue to use different classifications^11–14^, and some, including the ICE, use the classification of prematurity related deaths despite the recommendations from the WHO^8^. New classification systems have been proposed, yet no consensus has been reached^15,16^. In Sweden, death is certified in two stages: the Notification of Death and the Medical Death Certificate (MDC)^6^. Writing a MDC to define COD utilizes the WHO guidelines. A chronology of events leading to death is listed in the MDC, including the duration of each condition. The direct COD is defined as the final condition causing death^5,6^.

Unfortunately, there is a global trend for decreasing autopsy rates in infants^17^, which has also been seen in Sweden. A report from 2014 showed that infant autopsy rates had decreased from 71 to 35% for boys and from 64 to 31% for girls since 1987^7^. The former chief editor of JAMA George Lundberg warned in 1988 that autopsy reduction will negatively affect provided care, since lack of quality control of care will result in reduced knowledge of beneficial or negative effects of treatments^18^. Accurately defined COD reports are also needed for reliable and meaningful statistics. Records of preterm deaths are frequently incorrect, with flaws in the definition of live births, underreporting of births and deaths, and inaccurately stated CODs^19^. Some have advocated for postmortem MRI as a supplement to autopsies^20,21^. Although MRI brings some diagnostic value, it has been shown to miss more than half of the findings an autopsy would have provided, and neglects histological and microbiological features^20,21^.

The aim of this study was to determine the incidence of autopsy in preterm infants treated at our NICU between 2002 and 2018, and define how many of the preliminary CODs were revised by these autopsies. We also wanted to determine if there have been changes in the CODs over time. A better understanding of the significance of accurately determining the COD by autopsy in preterm infants may lead to the development of standardized protocols for defining COD.

## Results

The mortality for all premature infants born in Uppsala was 3.8% (n = 105/2763) during Period 1 and 5.0% (n = 160/3200) during Period 2 (*p* = 0.059). A total of 278 infants died during 2002–2018. No differences in birth weight, gestational age, degree of immaturity or age at death was found between the two periods (Table 1). There was a higher percentage of boys who died compared to girls in Period 2 (63.1% vs. 36.9%; *p* = 0.006; Table 1).

**Table 1:** Characteristics of deceased infants.

	Both periods	Period 1 2002–2009	Period 2 2011–2018	*p* value
**Population size**	265	105	160	–
**Gestational age, weeks—median (range)**	25 (21–36)	26 (21–36)	25 (21–36)	0.255
**Birth weight, g—median (range)**	742 (312–3970)	800 (312–3655)	710 (322–3970)	0.256
**Gestational age of those deceased—number (% of total in period)**				
32–36 weeks	37 (14.0)	16 (15.2)	21 (13.1)	0.627
28–31 weeks	51 (19.2)	19 (18.1)	32 (20.0)	0.700
< 28 weeks	177 (66.8)	70 (66.7)	107 (66.9)	0.972
**Distribution of sex—number (% of total in period)**				
Girls	109 (41.1)	50 (47.6)	59 (36.9)	0.265
Boys	156 (58.9)	55 (52.4)	101 (63.1)	0.373
Postnatal age at death in days—median (range)	7(0–308)	5 (0–239)	8 (0–308)	0.361
**Timing of death—number (% of total in period)**				
0–24 h	38 (14.3)	15 (14.3)	23 (14.4)	0.234
1–6 days	90 (34.0)	41 (39.0)	49 (30.6)	0.230
7–28 days	80 (30.2)	29 (27.6)	51 (31.9)	0.635
> 28 days, up to one year	57 (21.5)	20 (19.0)	37 (23.1)	0.429

Autopsy data are summarized in Table 2. The autopsy rate over the whole study period was 73.0% (n = 203/278), and did not change between the study periods (75% vs. 71%). Boys were autopsied in 70.2% of cases and girls in 76.9%. The main reason for an autopsy not being performed was parental refusal (n = 61/75; 81.3%). In 14.7% cases, there was no declared reason for autopsy not being performed. The frequency of autopsies was lower in those who died after 7 days of life (n = 96/141; 64.9%) as compared to those who died earlier (n = 107/137; 78.1%). Postmortem examinations changed the preliminary COD or confirmed the suspected COD in 35.0% (n = 71/203) of all autopsied cases (23.7% of the preliminary CODs were revised completely, and 11.3% of the suspected CODs were confirmed). In 5% (n = 10/203) of the autopsied cases it was not possible to define if autopsy altered the COD, due to lack of data on the preliminary COD. In Supplement 1, the preliminary COD and autopsy confirmed COD is listed for each individual where autopsy completely revised the COD. The CODs where autopsy enabled confirmation of a suspected COD are also listed in Supplement 1.

**Table 2:** Number of performed autopsies, diagnosis after autopsy of revised and confirmed suspected causes of death (CODs), and reasons for not performing autopsy (2002–2018).

	Number	Percent
**Number of performed autopsies**	203/278	73.0
Revised CODs after autopsy	48/203	23.6
Confirmed suspected CODs after autopsy	23/203	11.3
Not changed CODs after autopsy	112/203	55.2
Clinical CODs more precise than autopsy CODs*	10/203	4.9
Not available	10/203	4.9
**Revised and confirmed suspected CODs after autopsy**	71/203	34.9
Congenital anomaly	6/71	8.5
Asphyxia	1/71	1.4
Respiratory	13/71	18.3
IVH	4/71	5.6
Infection/sepsis	21/71	29.6
NEC	14/71	19.7
Other	12/71	16.9
**Reasons for not performing autopsy**	75/278	27.0
Parental refusal	61/75	81.3
Lack of medical indication	2/75	2.7
Other	1/75	1.3
Unknown	11/75	14.7

*If the autopsy defined the COD as “prematurity”, the clinical diagnosis was used as a definitive COD. In 3 of these 10 cases, the clinical COD was also prematurity.

### Cause of death: comparison between Period 1 and Period 2

**Figure 1:**
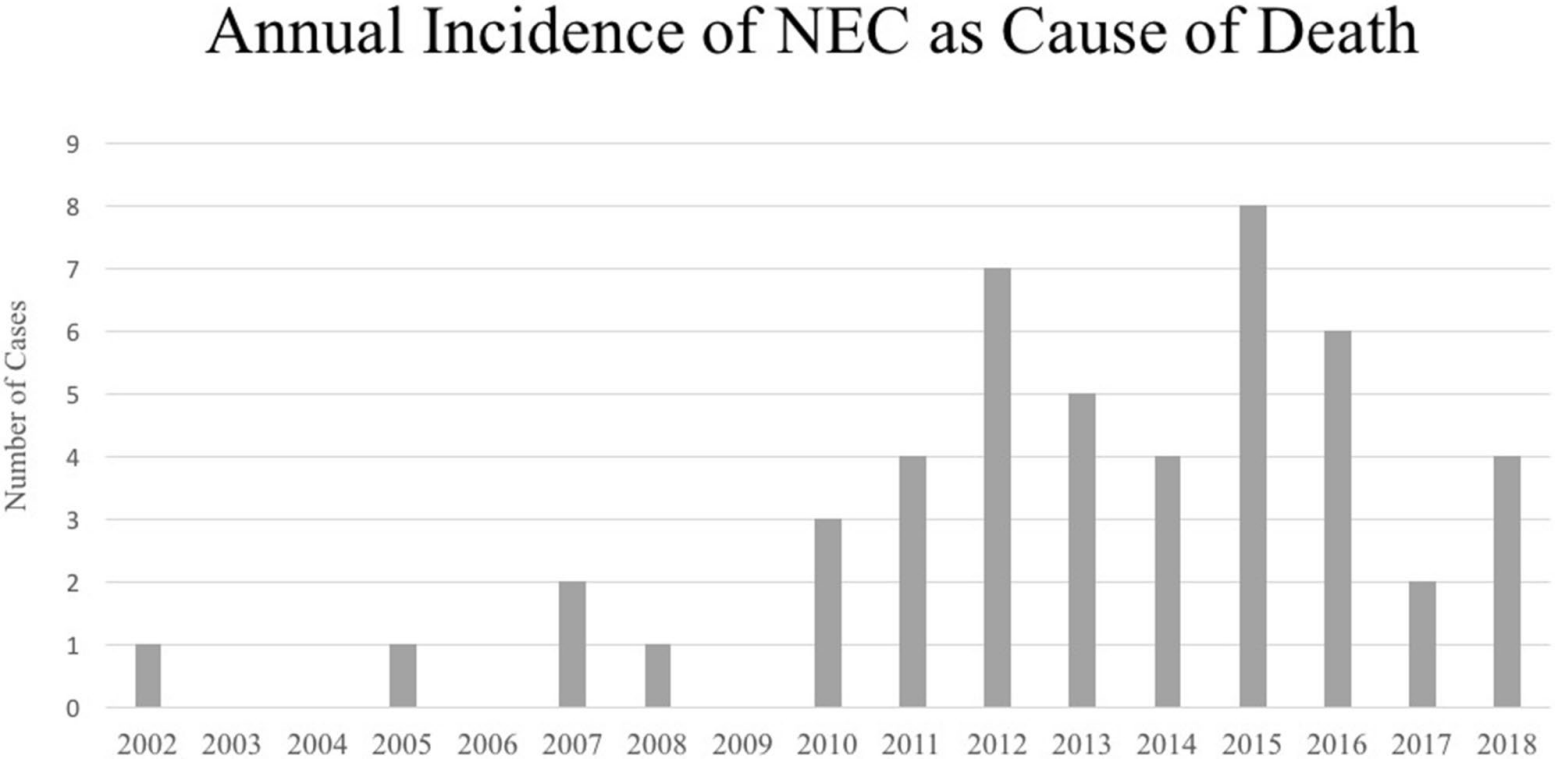
Annual incidence of NEC as cause of death from 2002 to 2018.

NEC as the COD increased from 5.0% (n = 5/101) to 25.2% (n = 40/159) (*p* < 0.001) from Period 1 to Period 2, whereas IVH as the COD decreased (*p* = 0.042) (Table 3). The annual incidence of NEC as a COD is shown in Fig. 1. Period 2 had fewer cases of RDS (*p* = 0.039) as COD than Period 1. The isolated spontaneous intestinal perforations (n = 4) were either consequences of anomalies or perforation without NEC. The type of infections as the COD during Period 1 and Period 2 was viral (n = 2), fungal (n = 2), bacterial pneumonia (n = 3) and systemic bacterial (n = 34).

**Table 3:** Cause of death in relation to year of birth.

	Both periods	Period 1 2002–2009	Period 2 2011–2018	*p* value
Total deaths (n)	265	105	160	–
Total deaths with determined cause of death (n)	260	101	159	–

Cause of death—number of cases (% of total)	n	n (%)	n (%)	
**Congenital anomalies**	18	10 (9.9)	8 (5.0)	0.132
Neural anomaly	3	2 (2.0)	1 (0.6)	0.562
Cardiac anomaly	3	2 (2.0)	1 (0.6)	0.562
Other anomaly	9	3 (3.0)	6 (3.8)	1.000
Chromosomal abnormalities	3	3 (3.0)	0 (0.0)	0.058
**Asphyxia**	17	10 (9.6)	7 (4.4)	0.080
Intrauterine	8	4 (4.0)	4 (2.5)	0.715
Perinatal	8	6 (5.9)	2 (1.3)	0.059
Postnatal	1	0 (0.0)	1 (0.6)	1.000
**Respiratory**	75	31 (30.7)	44 (27.3)	0.674
RDS	10	7 (6.9)	3 (1.9)	0.039
BPD	12	5 (5.0)	7 (4.4)	0.837
Pulmonary hypoplasia	20	8 (7.9)	12 (7.5)	0.912
PPHN	10	5 (5.0)	5 (3.1)	0.461
Pneumothorax	5	1 (1.0)	4 (2.5)	0.651
Haemothorax	4	3 (3.0)	1 (0.6)	0.302
Miscellaneous	14	2 (2.0)	12 (7.5)	0.087
**IVH**	22	13 (12.9)	9 (5.7)	0.042
**Infection/sepsis**	41	19 (18.8)	22 (13.8)	0.283
Early	11	3 (3.0)	8 (5.0)	0.537
Late	30	16 (15.8)	14 (8.8)	0.083
**NEC**	45	5 (5.0)	40 (25.2)	< 0.001
NEC with Sepsis	16	2 (2.0)	14 (8.8)	0.053
NEC without sepsis	29	3 (3.0)	26 (16.4)	0.001
**Other**	42	13 (12.9)	29 (18.2)	0.252
Shock/anemia/bleeding	8	1 (1.0)	7 (4.4)	0.156
Volvulus/malrotation	4	1 (1.0)	3 (1.9)	1.000
Isolated spontaneous intestinal perforation	4	0 (0.0)	4 (2.5)	0.160
Metabolic/electrolyte/endocrine disorders	4	2 (2.0)	2 (1.3)	0.643
Tumor	3	0 (0.0)	3 (1.9)	0.285
Twin-twin transfusion syndrome	2	2 (1.0)	1 (0.6)	0.562
CNS-related	3	1 (1.0)	2 (1.3)	1.000
Other	8	4 (4.0)	4 (2.5)	0.715
Prematurity	3	2 (2.0)	1 (0.6)	0.562
Unknown	2	0 (0.0)	2 (1.3)	0.523

### Cause versus timing of death

**Figure 2:**
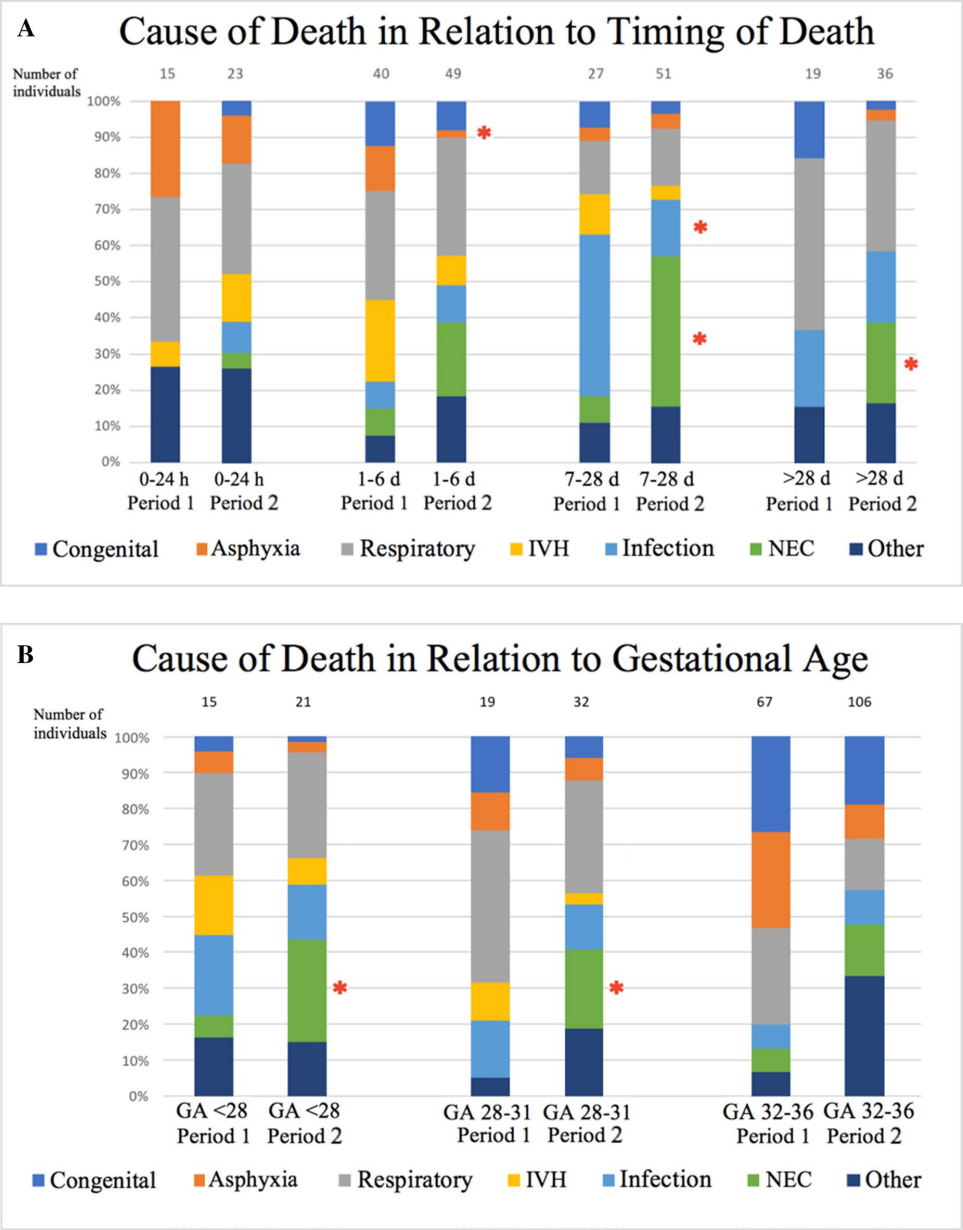
(**a**) Distribution in percent of causes of death at various time intervals (0–24 h; 1–6 days; 7–28 days; > 28 days) during Period 1 and Period 2. *p < 0.05; significant change between Period 1 and Period 2 within each time interval. Congenital, congenital anomalies; Respiratory, respiratory causes; IVH, intraventricular hemorrhage; Infection, infection with septicemia; NEC, necrotizing enterocolitis. See Table 4 for further categorization of causes of death. (**b**) Distribution in percent of causes of death at various degrees of immaturity (gestational age < 28 weeks, 28–31 weeks and 32–36 weeks) during Period 1 and Period 2. *p < 0.05; significant change between Period 1 and Period 2 within each degree of immaturity. GA; gestational age; Congenital, congenital anomalies; Respiratory, respiratory causes; IVH, intraventricular hemorrhage; Infection, infection with septicemia; NEC, necrotizing enterocolitis. See Table 4 for further categorization of causes of death.

As shown in Fig. 2a, fewer patients died due to perinatal asphyxia between day 1–6 (*p* = 0.050) in Period 2 than in Period 1. Cases with NEC as the COD increased for infants who died on days 7–28 and after 28 days (*p* = 0.001 and *p* = 0.025, respectively), while infection as the COD decreased (*p* = 0.006) between Period 1 and 2 for those infants who died on days 7–28. Median days of life for patients with NEC as cause of death was 4 (IQR = 8.5) in Period 1 and 11 (IQR = 11.5) in Period 2 (*p* = 0.023).

### Cause of death versus degree of immaturity

As presented in Fig. 2b, the incidence of NEC as COD in Period 2 was higher than in Period 1 for very preterm (*p* = 0.028) and extremely preterm infants (*p* < 0.001). There were no other significant differences in COD in the gestational age groups between Periods 1 and 2 (Fig. 2b).

## Discussion

In this study involving 278 preterms, we found an autopsy rate of 73% and a comparable distribution between sexes. The autopsy rate in this cohort was more than twice as high compared to national rates of infant autopsies in Sweden^7^. This might be explained by our local tradition of offering postmortem investigations at a regular basis to all parents in order to increase autopsy rates in preterm infants at UUCH, and thereby improving our knowledge of the COD. Infants who died at a higher postnatal age were autopsied less frequently than those who died at a lower postnatal age. Although unclear from this study, this difference in autopsy rates depending on postnatal age may be a consequence of there being more time to find a definitive diagnosis during a longer stay at the NICU, which is partly substantiated by de Séveaux et al. who found only minor additional findings with higher postnatal age at death^21^. Parents refusing to consent to autopsy was the main reason for autopsies not taking place, which is in agreement with previous findings^21,22^. Reduced parental consent may depend on the information provided by the health care regarding the procedure and potential benefit of an autopsy^22,23^. In many cases, the responsibility for obtaining consent for autopsy lies with the medical staff who may not fully understand the importance of the autopsy when discussing the procedure with the parents^22,23^. For the parents, the correct COD for their lost infant may be of importance for future pregnancy decisions, since genetic anomalies or syndromes can be difficult to diagnose solely on clinical examination of very preterm infants^21^.

Our main finding is that post-mortem examinations contributed considerably to determining the COD as evidenced by changing or confirming the suspected preliminary COD in a third of cases. This is consistent with results from previous studies, where a percentage of 25–36% CODs were found to be altered by autopsy in centers with generally low autopsy rates^21,22,24^. A higher autopsy rate might be expected to decrease the number of times the preliminary COD is revised, given that in studies with lower autopsy rates, the autopsy is performed when the COD is unclear to the clinician^17,21,25^. However, as shown in this study, even with relatively high autopsy rates the risk of changing the preliminary COD remains high. Thus, our data are consistent with the notion that improving autopsy rates will yield a higher quality of COD and therefore be more useful in understanding COD trends in the NICU.

The decrease of IVH as a COD between the two studied periods, is consistent with national findings in Sweden^26^ and may be attributed to more refined ventilatory strategies^27^ and more consistent treatment with antenatal corticosteroids^28^. Being a referral hospital for our region, the reduced rate of RDS as a COD could be due to the changed policy during Period 2 of referring all mothers at risk of delivering prematurely before 28 weeks GA to our hospital, an observation that needs further evaluation. We found that the incidence of congenital anomalies as a COD in our cohort was lower than in other comparable studies in high income countries^29^. One possible explanation could be consistent maternity care, improved prenatal screening for congenital anomalies and Swedish abortion practices. Another reason could be the targeting of a more precise COD in our study rather than applying a general diagnosis like congenital anomalies as the primary COD.

The occurrence of NEC as the COD was significantly higher during Period 2 as compared to Period 1. The increase of NEC was especially evident in extremely preterm infants and deaths during day 7–28 and after 28 days of life. This is likely due to NEC being a disease occurring after 1–2 of weeks of life^30^ and that the risk of NEC increases with decreasing gestational age^31^. Another potential factor in the overall increase of NEC as a COD could be that improvements in clinical care over time enable the infants to survive long enough to develop NEC, as has been suggested previously^32^. The median age at death for patients with NEC as COD increased significantly between Period 1 and Period 2. An increased incidence of NEC in Sweden has also been reported for these two time periods (2004–2007 vs. 2014–2016; 6% vs. 10%) with a simultaneously increased survival in the lowest gestational ages (70% vs. 77%)^33^. A lower reported incidence of surgically treated NEC from our unit to the Swedish Neonatal Quality Register in infants born between 22 and 27 weeks GA (22–24 GA, 5% vs. 7.5%; 25–27 GA, 1.5% vs. 3%)^26^, with similar to lower mortality rates, suggests that other factors than local case clustering of NEC from viral or bacterial outbreaks (so called epidemic NEC) contribute to NEC incidence in our study^34,35^. The increase in NEC as a COD cannot be only ascribed to more autopsies allowing more detailed diagnosis, since the autopsy rate was similar during both periods. Nor could any difference in breast-feeding strategies (same during both periods) or probiotic strategies (not introduced) explain this redistribution of CODs with higher NEC during the second period.

The retrospective observational study design seems relevant for this type of study, and seems to define the prevalence of different CODs and the value of autopsy in determining the definitive COD. However, given the observational design it is difficult to draw too strong conclusions regarding the causes for the observed changes in COD over time, and particularly for NEC, why further research is needed to explain these differences in COD. Structured national and international surveying of neonatal care is increasing worldwide^36^, and might have impact on understanding COD and changes in COD over time. Some other studies on infant mortality have systematically assigned prematurity as the major COD^13^, ignoring the recommendations of the WHO^8^. We found 10 cases in this cohort where prematurity was registered as the COD, however 7 of them could later be sorted into other categories using data from medical records and/or autopsies. Only in 3 out of 278 cases did prematurity remain as the final COD. The low rate of prematurity as COD demonstrates the feasibility of following the WHO recommendation regarding not assigning prematurity as the primary COD. Some of the complications of prematurity are difficult to diagnose in preterm infants, since symptoms may not be specific or may appear after a delay. In our study, nearly 75% of all diagnoses were autopsy-verified, substantiating the COD. All autopsies were performed by the same pathologist, which results in a high level of qualitative continuity in performing autopsies^22^. In addition, the neonatologist involved in the patient’s care usually attended the postmortem examination, thereby providing the pathologist with more detailed pre-mortem clinical findings, which facilitated the examination and supported the definitive COD.

This study highlights the importance of post-mortem examinations, which continue to yield valuable information and contribute to determine the definitive COD in preterm infants. Besides observing an increase in NEC and a decrease in IVH between the two periods, we found that the results from the autopsy revised or confirmed the suspected preliminary COD in a third of cases during the entire study period. Interestingly, 73% of all deceased preterm infants during this period were autopsied and not only the ones with uncertain diagnoses. Our findings suggest that it would be beneficial to reverse the negative trend in autopsy rates in preterm infants, in order to obtain reliable COD data.

## Methods

### Study setting and ethical consideration

This study is a retrospective observational study of all preterm deaths from 2002 to 2018 and a 2-period sub-analysis (2002–2009 vs. 2011–2018) in a tertiary NICU at Uppsala University Children’s Hospital (UUCH). In order to exclude overlap of early and late deaths, and seasonal variations in COD, 2010 was excluded from the 2-period analysis. The study has been approved by the Regional Ethical Review Board of Uppsala, D:nr 2019-05033, and by the Uppsala Biobank, D:nr BbA-827-2019-094. With these endorsements infants were legally approved to be included in this study. Parental informed consent to perform an autopsy was obtained. All methods in this study were performed in accordance with relevant operating guidelines and regulations. Data were de-identified before analysis, according to operative ethical regulations.

### Inclusion of participants

**Figure 3:**
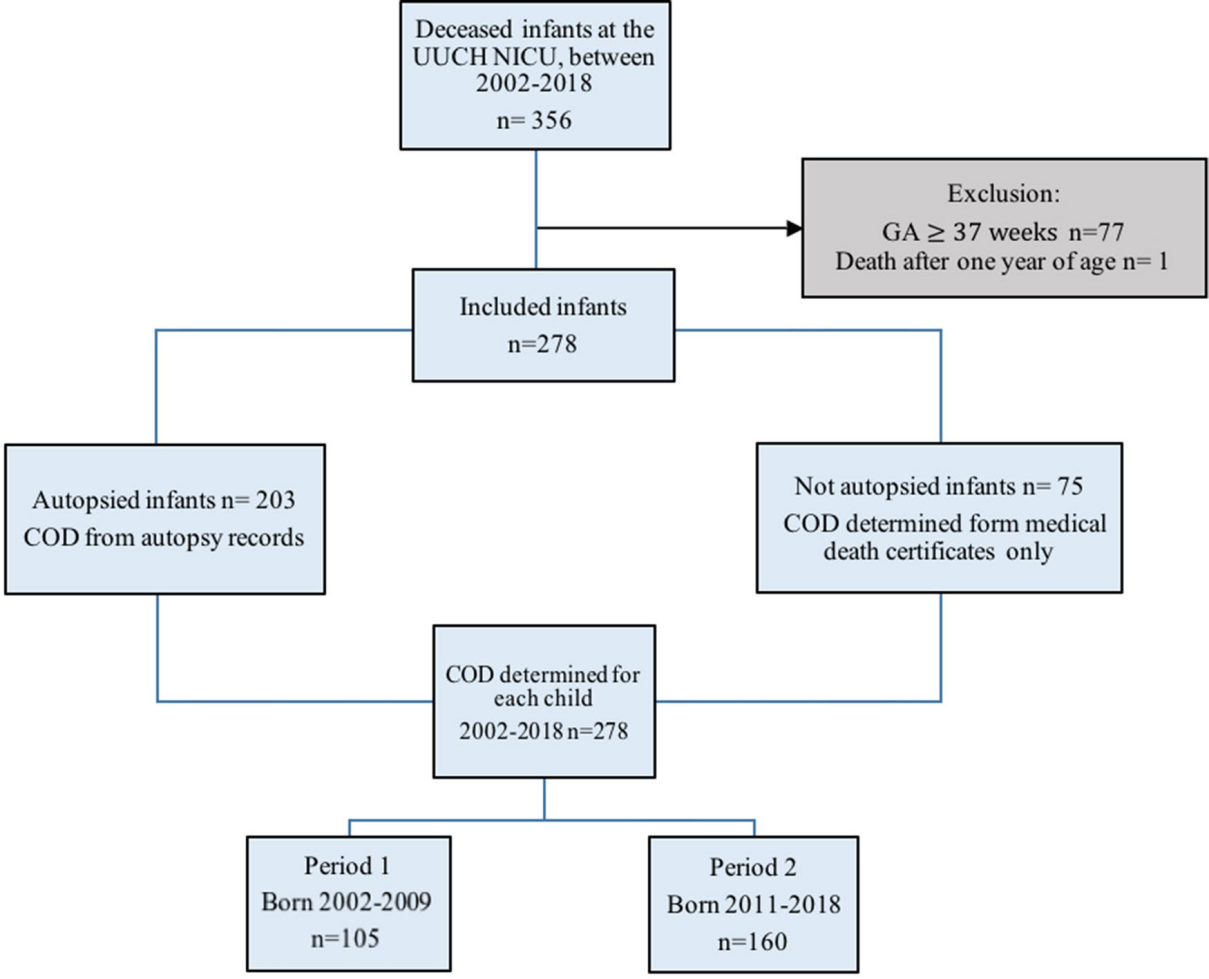
Inclusion of participants, data extraction and period division.

Figure 3 shows a flowchart of the selection of participants. All live born infants admitted to our NICU who subsequently died during the period of 2002 to 2018 were extracted from the Swedish Neonatal Quality Registry (SNQ) (n = 356). To study solely infant mortality, patients who died after 1 year of age were excluded (n = 1). Term infants (≥ 37 weeks gestation) were excluded (n = 77). Thus, there were a total of 278 infants that met inclusion criteria and had none of the exclusion criteria.

For the comparison between preliminary COD and definitive COD according to autopsy, there were 203 infants with an autopsy (Fig. 3). Medical Death Certificate (MDCs) were examined to determine preliminary cause of death. If multiple MDCs were issued, the MDC issued prior to autopsy was used to avoid the preliminary COD being influenced by autopsy findings. If the MDC was issued after autopsy findings, the discharge note was examined to determine the presumed COD prior to autopsy. MDC and the discharge note were written by the attending physician well acquainted with the patient, and confirmed by the head physician in charge of the ward. An autopsy included a macroscopic review of tissues with a neonatologist present, immune-histological analysis linked to symptomology, bacterial cultures and PCR-analysis if suspicion for viral infection from clinical symptoms or from macroscopic findings was present. The present neonatologist provided clinical data, contributing to the definitive cause of death as determined by autopsy. All infant autopsies were performed by the same pathologist and none was restricted in scope. Parental informed consent was required for autopsy. All parents are offered autopsy by the attending physician on a regular basis, to define the definitive COD and/or other diseases relevant for the course of care and for future pregnancies. For the individuals who were not autopsied, the reason for an autopsy not being performed was defined.

### Data extraction and study variables

Perinatal and postnatal data were collected from the SNQ. Perinatal data included time of birth, GA, sex, and birth weight. GA was documented as complete weeks and days, determined by ultrasound. Postnatal data included whether autopsy was performed or not, days of life, and if recorded, why an autopsy was not performed. If the autopsy was not performed due to parental reluctance no retrospective inquiry was performed, since this was considered ethically inappropriate. Missing data in SNQ were complemented by retrieving Medical Death Certificates (MDC) and charts from Cosmic (electronic journal database after 2008) or from Regional archives (before 2008). Autopsy records were collected from the pathology chart system Sympathy. Premature birth rates for the Uppsala Region were obtained from SNQ. Cause of death was determined according to ICD-10 codes^8^. Definitive COD was determined from autopsy records for those who underwent autopsy (n = 203). For the rest (n = 75), MDCs and discharge notes were used for definitive COD. Causes of death were categorized by groups based on ICE^10^ and further refined in alignment with categories used in recent publications^11–13^. The final categories are presented in Table 4. The categories were established prior to data collection, to avoid the categorization being influenced by the data.

**Table 4:** Categories for causes of death.

**Congenital anomalies**	**Intraventricular hemorrhage**
Neural anomaly	**Infection/sepsis**
Cardiac anomaly	Infection/sepsis, early
Other anomaly	Infection/sepsis, late
Chromosomal abnormalities	**Necrotizing enterocolitis (NEC)**
**Asphyxia**	NEC without sepsis
Intrauterine	NEC with sepsis
Perinatal	**Other**
Postnatal	Shock/anemia/bleeding
**Respiratory causes**	Volvulus/malrotation
Bronchopulmonary dysplasia	Isolated spontaneous intestinal perforation
Respiratory distress syndrome	Metabolic/electrolyte/endocrine disorders
Pulmonary hypoplasia	Tumor
Persistent pulmonary hypertension	Twin-to-twin transfusion syndrome
Pneumothorax	CNS-related
Haemothorax	Other
Miscellaneous	Prematurity

For autopsied infants, MDCs were examined to compare preliminary and definitive COD. If the MDC was issued after autopsy findings, the discharge note was examined to determine the presumed COD prior to autopsy. The preliminary COD and the definitive COD were compared to see if the definitive COD was altered due to information arising from the autopsy. The outcome after autopsy could be one of three: no change of COD; revised COD; or confirmed suspected but not yet established COD. Suspected COD could for instance be a suspected infection, but without positive cultures or identification of the location of the infection. If COD from the autopsy was “Prematurity” (n = 10), but the clinicians found a more precise COD, the more precise COD was used as the definitive COD, in line with the recommendations from the WHO^8^.

### Statistical analyses

Descriptive statistics were calculated in Excel, Version 15.27 (161010). The rest of the statistical analysis was performed in SPSS version 26. A *p* value < 0.05 was considered significant. Comparisons between groups for continuous data were done using the Mann–Whitney U-test. For trend difference analysis in COD from 2002 to 2009 (n = 105) and 2011–2018 (n = 160), both autopsied and not autopsied infants were included. A chi-square test was used to compare the frequency of each diagnosis between the two periods, and complemented with a Fisher’s exact test if less than 5 occurrences were observed. The comparison between preliminary COD and definitive COD according to autopsy was performed on autopsied individuals from 2002 to 2018 (n = 203).

## Supplementary Information

Supplementary Information.

## Data Availability

All data generated or analyzed during this study are included in this published article (and its Supplementary Information files).

## Abbreviations

BPD: Bronchopulmonary dysplasia
COD: Cause of death
IVH: Intraventricular hemorrhage
MDC: Medical death certificate
NEC: Necrotizing enterocolitis
RDS: Respiratory distress syndrome
SNQ: Swedish neonatal quality registry
UUCH: Uppsala University Children’s Hospital

## References

[1] Chawanpaiboon S, Vogel JP, Moller A-B, Lumbiganon P, Petzold M, Hogan D (2019). Global, regional, and national estimates of levels of preterm birth in 2014: a systematic review and modelling analysis. Lancet Glob. Health.

[2] GBD 2016 Causes of Death Collaborators (2017). Global, regional, and national age-sex specific mortality for 264 causes of death, 1980–2016: a systematic analysis for the global burden of disease study 2016. Lancet.

[3] Blencowe H, Cousens S, Oestergaard MZ, Chou D, Moller A-B, Narwal R (2012). National, regional, and worldwide estimates of preterm birth rates in the year 2010 with time trends since 1990 for selected countries: a systematic analysis and implications. Lancet.

[4] Petrou S, Henderson J, Bracewell M, Hockley C, Wolke D, Marlow N (2006). Pushing the boundaries of viability: the economic impact of extreme preterm birth. Early Hum. Dev..

[5] World Health Organization (2011). ICD-10: International statistical classification of diseases and related health problems.

[6] Brooke HL, Talbäck M, Hörnblad J, Johansson LA, Ludvigsson JF, Druid H (2017). The Swedish cause of death register. Eur. J. Epidemiol..

[7] National Board of Health and Welfare. Causes of Death 2014 [Internet]. 2015 https://www.socialstyrelsen.se/globalassets/sharepoint-dokument/artikelkatalog/statistik/2015-8-1.pdf. Accessed 3 Oct 2019.

[8] World Health Organization. The WHO application of ICD-10 to deaths during the perinatal period: ICD-PM [Internet]. World Health Organization; 2016 https://apps.who.int/iris/bitstream/handle/10665/249515/9789241549752-eng.pdf?sequence=1. Accessed 15Oct 2019.

[9] Wigglesworth JS (1980). Monitoring perinatal mortality. A pathophysiological approach. Lancet.

[10] Alberman E, Bergsjø P, Cole S, Evans S, Hartford R, Hoffman H (1989). International Collaborative Effort (ICE) on birthweight; plurality; and perinatal and infant mortality. I: Methods of data collection and analysis. Acta Obstet. Gynecol. Scand..

[11] Berrington JE, Hearn RI, Bythell M, Wright C, Embleton ND (2012). Deaths in preterm infants: changing pathology over 2 decades. J. Pediatr..

[12] Park JH, Chang YS, Sung S, Ahn SY, Park WS (2017). Trends in overall mortality, and timing and cause of death among extremely preterm infants near the limit of viability. Gay N, editor. PLoS ONE.

[13] Patel RM, Kandefer S, Walsh MC, Bell EF, Carlo WA, Laptook AR (2015). Causes and timing of death in extremely premature infants from 2000 through 2011. N. Engl. J. Med..

[14] Stoll BJ, Hansen NI, Bell EF, Walsh MC, Carlo WA, Shankaran S (2015). Trends in care practices, morbidity, and mortality of extremely preterm neonates, 1993–2012. JAMA.

[15] Dias BAS, Santos ETD, Andrade MAC (2017). Classification systems for avoidability of infant deaths: different methods, different repercussions?. Cad Saude Publica.

[16] Nakamura AM, Dove MS, Minnal A, Damesyn M, Curtis MP (2015). Infant mortality: development of a proposed update to the Dollfus classification of infant deaths. Public Health Rep..

[17] Bassat Q, Castillo P, Alonso PL, Ordi J, Menéndez C (2016). Resuscitating the dying autopsy. PLoS Med..

[18] Lundberg GD (1988). Now is the time to emphasize the autopsy in quality assurance. JAMA.

[19] Paulson J, Ramsini W (2008). Unregistered deaths among extremely low birthweight infants—Ohio, 2006. JAMA.

[20] Cohen MC, Paley MN, Griffiths PD, Whitby EH (2008). Less invasive autopsy: benefits and limitations of the use of magnetic resonance imaging in the perinatal postmortem. Pediatr. Dev. Pathol..

[21] de Sévaux JLH, Nikkels PGJ, Lequin MH, Groenendaal F (2019). The value of autopsy in neonates in the 21st century. Neonatology.

[22] Elder DE (2005). Autopsy after death due to extreme prematurity. Arch. Child. Fetal Neonatal Ed..

[23] Mjörnheim B, Rosendahl A, Eriksson L (2015). Attitudes of nurses and physicians about clinical autopsy in neonatal and adult hospital care: a survey in Sweden. Nurs. Res..

[24] Killeen OG, Burke C, Devaney D, Clarke TA (2004). The value of the perinatal and neonatal autopsy. Ir. Med. J..

[25] Seske LM, Muglia LJ, Hall ES, Bove KE, Greenberg JM (2017). Infant mortality, cause of death, and vital records reporting in Ohio, United States. Matern. Child Health J..

[26] SNQ Report Group. Årsrapport 2018, Yearly Report 2018 [Internet]. 2019 Aug https://www.medscinet.com/PNQ/uploads/website/arsrapporter/Neonatalregistrets%20%C3%85rsrapport%202018._3.0.pdf. Accessed 11 Oct 2019.

[27] Klingenberg C, Wheeler KI, McCallion N, Morley CJ, Davis PG (2017). Volume-targeted versus pressure-limited ventilation in neonates. Cochrane Database Syst Rev..

[28] Roberts D, Brown J, Medley N, Dalziel SR (2017). Antenatal corticosteroids for accelerating fetal lung maturation for women at risk of preterm birth. Cochrane Database Syst. Rev..

[29] Lehtonen L, Gimeno A, Parra-Llorca A, Vento M (2017). Early neonatal death: a challenge worldwide. Semin. Fetal Neonatal Med..

[30] Rasiah V, Yajamanyam PK, Ewer AK (2014). Necrotizing enterocolitis: current perspectives. Res. Rep. Neonatol..

[31] Samuels N, van de Graaf RA, de Jonge RCJ, Reiss IKM, Vermeulen MJ (2017). Risk factors for necrotizing enterocolitis in neonates: a systematic review of prognostic studies. BMC Pediatr..

[32] Travers CP, Clark RH, Spitzer AR, Das A, Garite TJ, Carlo WA (2017). Exposure to any antenatal corticosteroids and outcomes in preterm infants by gestational age: prospective cohort study. BMJ.

[33] Norman M, Hallberg B, Abrahamsson T, Björklund LJ, Domellöf M, Farooqi A (2019). Association between year of birth and 1-year survival among extremely preterm infants in Sweden during 2004–2007 and 2014–2016. JAMA.

[34] Bagci S, Eis-Hübinger AM, Franz AR, Bierbaum G, Heep A, Schildgen O (2008). Detection of astrovirus in premature infants with necrotizing enterocolitis. Pediatr. Infect. J..

[35] Meinzen-Derr J, Morrow AL, Hornung RW, Donovan EF, Dietrich KN, Succop PA (2009). Epidemiology of necrotizing enterocolitis temporal clustering in two neonatology practices. J. Pediatr..

[36] Kiechl-Kohlendorfer U, Simma B, Urlesberger B, Maurer-Fellbaum U, Wald M, Wald M (2019). Low mortality and short-term morbidity in very preterm infants in Austria 2011–2016. Acta Paediatr..

