# Supplementary material for: The value of autopsy in preterm infants at a Swedish tertiary neonatal intensive care unit 2002–2018
Source: Sci Rep. 2021 Jul 8;11:14156. doi: 10.1038/s41598-021-93358-7 (PMC8266827; doi:10.1038/s41598-021-93358-7)
Supplement: Supplementary file 1 — Supplementary Information. [file 41598_2021_93358_MOESM1_ESM.pdf]

The Value of Autopsy in Preterm Infants at a Swedish Tertiary Neonatal Intensive Care Unit 2002-2018

Alice Hoffsten MD[1], Laszlo Markasz MD PhD[1,2], Katharina Ericson MD[3], Leif D Nelin MD [1,4] and Richard Sindelar MD PhD[1,2]

[1]Department of Women's and Children´s Health, Uppsala University, Uppsala, Sweden

[2]Neonatology Division, Uppsala University Children's Hospital, Uppsala, Sweden

[3]Department of Pathology, Uppsala University Hospital, Uppsala, Sweden

[4]Nationwide Children's Hospital, Ohio State University College of Medicine, Columbus, Ohio, USA

Supplement 1

Suspected Cause of Death	Definite Cause of Death Found by Autopsy
Infection/Sepsis (Early)	Respiratory (BPD)
Congenital anomaly (Other anomaly)	Congenital anomaly (Neural anomaly)
Other (Other anomaly)	Respiratory (RDS)
Infection/Sepsis (Early, suspected)	Other (Other anomaly)
Infection/Sepsis (Early, suspected)	Respiratory (RDS)
Other (Metabolic)	NEC + Sepsis
Respiratory (Miscellaneous)	Infection/Sepsis (Late)
Other (Other anomaly)	Congenital (Other anomaly)
Other (Shock/anemia/bleeding)	Infection/Sepsis (Late)
Respiratory (Other)	Respiratory (Pulmonary Hypoplasia)
Infection/Sepsis (Late)	NEC
Respiratory (Other)	Congenital (Other anomaly)
NEC (Suspected)	Other (premature)
Respiratory (Pneumothorax)	Other (TTTS)
Respiratory (Pneumothorax)	Other (TTTS)
Respiratory (Pulmonary hypoplasia)	Respiratory (RDS)
Other (Isolated spontaneous intestinal perforation)	Infection/Sepsis (Late)
Infection/Sepsis (Late)	Other (Volvulus/malrotation)
Other (Metabolic)	IVH
Asphyxia (Perinatal)	Respiratory (RDS)
Other (Isolated spontaneous intestinal perforation)	NEC
Infection/Sepsis (Late)	NEC + Sepsis
Infection/Sepsis (Late, suspected)	Infection/Sepsis (Early)
Congenital (Chromosomal abnormality)	Respiratory (PPHN)
Respiratory (Pulmonary hypoplasia)	Congenital (Other anomaly)
Congenital (Other anomaly)	Infection/Sepsis (Early)
Asphyxia (Perinatal)	NEC
Respiratory (BPD)	Respiratory (PPHN)
Other (Metabolic/electrolyte/endocrine disorders)	NEC
Other (Prematurity)	Infection/Sepsis (Early)
Other (Other anomaly)	Infection/Sepsis (Late)
Respiratory (Hemothorax)	NEC + Sepsis
Infection/Sepsis (Late)	NEC
Respiratory (Pulmonary hypoplasia)	Respiratory (Miscellaneous)
Respiratory (Hemothorax)	Infection/Sepsis (Early)
Other (TTTS)	Other (Shock/anemia/bleeding)
Asphyxia (Perinatal)	Other (Shock/anemia/bleeding)
Other (Volvulus/malrotation)	Congenital (Other anomaly)
Other (Chock/anemia/bleeding)	Infection/Sepsis (Early)
Respiratory (RDS)	IVH
Other (Metabolic/electrolyte/endocrine disorders)	Other (Shock/anemia/bleeding)
Respiratory (PPHN)	Other (Shock/anemia/bleeding)

Infection/Sepsis (Early)	Other (Isolated spontaneous intestinal perforation)
Asphyxia (Intrauterine)	Respiratory (Pulmonary hypoplasia)
Respiratory (Pulmonary hypoplasia)	Other (TTTS)
Other (Isolated spontaneous intestinal perforation)	Infection/Sepsis (Late)
Infection/Sepsis (Late, suspected)	NEC + Sepsis
Infection/Sepsis (Late)	NEC + Sepsis

**Suspected Cause of Death Confirmed by Autopsy**
NEC
Infection/Sepsis (Late)
Infection/Sepsis (Late)
Infection/Sepsis (Late)
Infection/Sepsis (Late)
Infection/Sepsis (Late)
Respiratory (Pulmonary Hypoplasia)
Other (Other anomaly)
Infection/Sepsis (Late)
NEC+ Sepsis
NEC
Congenital (Other anomaly)
Infection/Sepsis (Early)
IVH
Infection/Sepsis (Late)
Respiratory (Miscellaneous)
NEC
Infection/Sepsis (Late)
IVH
Respiratory (Pulmonary hypoplasia)
Infection/Sepsis (Late)
Asphyxia (Intrauterine)
Infection/Sepsis (Late)

BPD, Bronchopulmonary Disease; RDS, Respiratory Distress Syndrome; NEC, Necrotizing Enterocolitis; TTTS, Twin-to-Twin Transfusion Syndrome; IVH, Intraventricular Hemorrhage; PPHN, Persistent Pulmonary Hypertension of the Newborn,
